# Charting New Frontiers for Black Men's Brain Health: Implications for Dementia Research and Public Engagement

**DOI:** 10.1002/hast.4996

**Published:** 2025-09-18

**Authors:** Darlingtina K. Esiaka

**Keywords:** Black men's health, brain health, dementia research, health equity, community engagement, social determinants of health, bioethics

## Abstract

This essay critically explores the intersection of brain health and dementia research within the context of Black men's lived experiences. It interrogates dominant narratives of brain health that prioritize individual responsibility while neglecting systemic and structural determinants of health. Drawing on a life‐course perspective, the essay advocates for inclusive research practices that center Black men's voices, challenge deficit‐based assumptions, and address systemic barriers to brain health. Finally, it outlines a roadmap for transformative research, advocacy, and care that advances brain health equity.

In recent years, I have become intrigued by the concept of *brain health*—the optimal state of brain functioning that encompasses its cognitive, sensory, social‐emotional, behavioral, and motor aspects.^1^ Over the years, the concept of brain health for older adults has been used in promoting healthy aging and addressing the risk of cognitive decline in a more positive and proactive light.^2^ Within the broader discourse of healthy aging, narratives about brain health can be empowering. They can emphasize individual responsibilities and illuminate preventative approaches that individuals can use to maintain and support cognitive function and well‐being in older age^3^ and reduce the risk of dementia, such as engaging in physical and intellectually stimulating activities, maintaining a healthy lifestyle, and participating in social interactions.^4^

However, it is essential to ensure that these narratives focused on individual choice do not inadvertently downplay or oversimplify the complexities of cognitive health and the risk of cognitive decline in aging populations that bear a disproportionately negative impact from the systemic factors, such as inequitable access to health care, that are among the root causes of poor brain health. I am particularly interested in understanding pathways to brain health within communities facing stark risks of dementia and in promoting a narrative of brain health that is reflective of the lived experiences of Black men. This interest is spurred by the crucial and urgent need for culturally relevant strategies to improve brain health while addressing the unique risk factors for poor brain‐health outcomes in this population. Brain health among Black men is shaped by complex factors, including structural racism, systemic barriers to health care in general and mental health care in particular, and socioeconomic disparities; improving brain health in this population requires cultural narratives and practical approaches that address cognitive well‐being and dementia risk with sensitivity, acknowledging the diverse experiences within Black communities and working toward creating a more inclusive and equitable concept of brain health. Narratives that support these goals recognize that Black men's brain‐health outcomes are not solely the result of individual choices but are deeply influenced by historical and ongoing inequities.

This essay reflects my past and ongoing involvement with several important initiatives, including the Aging and Brain Health Alliance at Rutgers University; Black Men's Brain Health; Alter Dementia; the Michigan Center for Contextual Factors in Alzheimer's Disease; the HBCU Aging Conference; Empucate International; the Louisville, Kentucky, chapter of the African American Male Wellness Agency; and the Black in Gerontology and Geriatrics Network. These initiatives are directed toward achieving equity and justice in health outcomes and Alzheimer disease and related dementias in Black communities.

As a Black immigrant woman with over a decade of experience studying Black men's health in the United States and West Africa, I acknowledge the unique lens through which I approach my research and thus this essay. My positionality is shaped by my lived experiences as a Black woman in a society that has historically and continuously marginalized and oppressed Black people. I recognize that Black men face unique challenges and experiences that differ from mine, and it is important for me to acknowledge and respect their perspectives in my research. My positionality also influences my research questions, methodology, and analyses, helping me to recognize the importance of centering the voices and experiences of Black men in my research, as their perspectives have often been overlooked or marginalized in academic research. I aim to approach my research with humility, respect, and a willingness to learn, and I am committed to centering the voices and experiences of Black men in my work.

## Dementia in Black Americans

Older Black Americans are disproportionately burdened by Alzheimer disease and related dementias compared to older White Americans. Compared to their White counterparts, they are over twice as likely to develop dementia.^5^ Dementia is the third leading cause of death in non‐Hispanic Black men as a whole and the fourth leading cause of death in those eighty‐five or older.^6^ Despite living in one of the wealthiest, most technologically and medically advanced countries, Black men in the United States have an average health status similar to that of men in low‐ and middle‐income countries such as El Salvador, Iran, and Vietnam.^7^ In 2011, David Williams, a scholar of public health, described the health of Black men as an all‐pervasive “crisis”—a term he used to characterize the noticeably increased rates of disability, morbidity, and mortality of Black men compared with White men.^8^

A careful look at past studies done primarily in White Americans provides evidence that men have a higher risk of mild cognitive impairment^9^ and more rapid progression from mild cognitive impairment to dementia.^10^ However, it is unclear if this is the case for Black men. Black Americans, particularly Black men, have over the years shown low interest in participating in dementia research.^11^ How can researchers fully understand the mechanisms of dementia across diverse American populations when Black men are not represented in dementia research, including clinical trials? Given the Black community's well‐warranted distrust toward medical research, how do researchers initiate and sustain conversations about brain health and research participation with Black men?

Efforts to advance brain health and dementia research among Black men must begin and end with involving and centering Black men. Below, I discuss some important lessons from my involvement with initiatives targeting Black men that I believe, when widely applied by researchers, can chart new frontiers for the brain health of aging Black men, ensure that dementia research efforts benefit Black men, and promote a narrative of brain health reflective of the lived experiences of Black men.

## Myths about Black Men's Health

Research reveals several myths that purport to explain Black men's health. According to these explanatory myths, which may feature in cultural narratives, Black men are inherently unhealthy, have poor health behaviors, and are at a high risk of certain health conditions solely because they are Black.^12^ These myths can influence the ways in which dementia researchers study Black men. I propose that any efforts made toward improving the narratives surrounding brain health must first acknowledge the influence of these myths on dementia research. These myths perpetuate negative stereotypes and contribute to health disparities by ignoring the lived experiences of Black men within structures that impact their health outcomes, such as systems that promote poverty and impose barriers to opportunity and health care.^13^ Researchers, clinicians, public health workers, and others could be better able to promote brain health if they recognized that, despite the low expectations from society, Black men strive for good health and are interested in participating in dementia research if they feel safe and empowered.^14^

When researchers engage Black men, they can gain the knowledge necessary for recognizing unique issues contributing to Black men's brain health. One such issue that typically sets Black men on a path to experiencing poorer health outcomes is the Black male tax. “Black male tax” refers to the nuanced day‐to‐day responsibilities of Black men that encourage them to place the needs of their families and other community members above their own needs, while being constantly alert to how they are being viewed and judged within society. With the Black male tax, the onus is on Black men to ensure the survival of their families, communities, and the Black race. To improve brain health among Black men, researchers must examine how the cumulative demands of the Black male tax and other structural stressors shape health behaviors and neurological well‐being. Identifying and reducing barriers to the inclusion of Black men in dementia research is also integral to changing the narrative of Black men's health so that it is a more useful and supportive narrative for this population. As researchers, we must ensure that these shifts involve clinicians, policy‐makers, community leaders, and Black men.

## Early Detection of Cognitive Decline and Identification of Modifiable Risk Factors

Charting a new frontier for Black men's brain health involves early detection of cognitive decline and identification of modifiable risk factors long before the recommended age for dementia screening. Older age is a key risk factor for dementia in all racial and ethnic groups. However, it is widely acknowledged that dementia can have various causes and risk factors, and the age of onset can vary significantly depending on dementia type and on individual factors such as genetics, lifestyle, and medical history.^15^ In addition, age‐related decline begins earlier in men than in women.^16^ Data consistently show that Black men have the lowest life expectancy of any major demographic group in the United States, excluding Indigenous groups.^17^ Black men are thus more likely to die before old age, when dementia is commonly screened for and diagnosed.

However, Black men are also at higher risk for chronic illnesses such as hypertension and diabetes and for head trauma, all of which are precursors for cognitive decline to dementia; they are also at higher risk for early onset of hypertension and diabetes.^18^ Resources such as Cognition in Primary Care, a toolkit and training program to facilitate the early detection of cognitive impairment in the most common health care setting for older adults, are now available to help practitioners identify modifiable risk factors as well as early symptoms.^19^ Research shows that up to 45 percent of dementia cases may be attributable to modifiable factors such as physical inactivity, depression, smoking, and excessive alcohol use, which disproportionately affect Black men and contribute to their elevated risk of dementia.^20^

Dementia screening is typically recommended for people sixty‐five years of age and older. Screening Black men earlier than sixty‐five would be a practical response to their elevated risks of both dementia and early‐onset precursor conditions and would enable practitioners to collaborate with these patients to begin risk‐reduction modifications. New frontiers in Black men's brain health therefore include improved communication between patients, family members, and practitioners in primary care settings and collaborations between research participants, researchers, and practitioners to develop and implement effective interventions that can reduce dementia risk factors, potentially improving outcomes and extending brain health, while also reducing health disparities between Black men and other older adults.

The importance of early detection of cognitive decline and the identification of modifiable risk factors is underscored by the reality that the era of effective pharmacological interventions for dementia has not yet arrived. There are currently very few approved drugs, with no long‐term data on their effectiveness on non‐Hispanic White populations.^21^ These drugs carry high burdens and risks and may not improve a person's quality of life.^22^ Existing drugs seem to be effective only at very early‐stage disease, yet waiting lists at memory‐care clinics can be a year long, and minoritized Americans are often diagnosed at later stages of dementia progression.^23^ Broader recognition by dementia researchers that earlier detection of cognitive decline and identification of modifiable risk factors can take place in the primary care setting will help ensure that Black men and their families receive timely and effective care and receive support for navigating the impacts of a dementia diagnosis, such as stigma, financial burden, and caregiving.

## Increasing Dementia Awareness through Relationship Building and Partnerships

Additional efforts are needed to increase awareness of the importance of brain health and the unique challenges that Black men face.^24^ Specifically, efforts to change the reality of Black men's health must include building relationships with Black men and involving them equally with researchers at every stage of the research process. This can be achieved using community‐engaged research.^25^ This type of research can ensure that a research process is scientifically rigorous, grounded in the lived experiences of older Black men, and aligned with community needs. A community‐engaged research practice recognizes the importance of relationships and can help researchers avoid blind spots. As evidenced by initiatives such as the Alzheimer's awareness program for Black men in Newark, New Jersey, supported by the New Jersey Health Foundation and championed by the Aging and Brain Health Alliance during my time at Rutgers University, these efforts place Black men at the center of the discussion about Black men's brain health.^26^

For better outcomes for Black men and for Black men to actively participate in dementia research, including clinical trials, researchers must present them with opportunities to advocate for themselves. For example, the Black Men's Brain Health (BMBH) initiative, led by Robert W. Turner II, Monica Rivera‐Mindt, and Maria C. Carrillo, connects scientists, practitioners, and community partners around the goals of increasing the inclusion of Black men in brain science research and improving brain health among Black men. The BMBH initiative, which includes the BMBH directory for Black men interested in learning about research studies, models an effective and sustainable process of engaging Black men as partners and building trust. This process includes presenting the BMBH initiative as trustworthy by maintaining transparency in communication, involving Black men in decision‐making roles, and ensuring cultural relevance in all outreach efforts. For example, the initiative partners with trusted community organizations and hosts events in familiar, accessible spaces during the Superbowl. Trust is reinforced by identifying mutual goals, delivering on promises, and providing a platform for the next generation of scholars interested in researching brain health in Black men. Each year, the BMBH team holds a conference to raise awareness about brain science research among Black men. The conference presents opportunities for relationship formation and partnerships with Black men. I was one of the 2022 inaugural BMBH conference scholars, who were provided with resources and funding to conduct research focused on Black men. The conference fosters an environment where scholars, established researchers, and community partners are encouraged to critically examine prevailing narratives and engage in an iterative process aimed at reshaping how Black men's brain health is understood, represented, and addressed in research and public discourse.

## A Holistic Approach to Changing the Narrative of Black Men's Brain Health

Attempts to improve the status of Black men's brain health should not rely on narratives that promote the idea that this goal depends on individual factors alone or that it depends on societal factors alone.^27^ A holistic approach recognizes the complex issue of promoting brain health among Black men by balancing attention to, on the one hand, the efforts the men make as individuals and, on the other hand, the impact of social determinants on their health. Some researchers may ignore the presence of racialized disadvantages related to health equity and justice, such as food insecurity, neighborhood disadvantages, poor job opportunities, poverty, and structural racism, and attribute responsibility for brain health outcomes among Black men solely to these men. For example, Black men may be seen as solely responsible for promoting their own brain health through personal actions such as exercising, eating healthily, sleeping well, and other strategies. However, social determinants of health, grounded in the circumstances in which individuals are born, grow up, live, work, and age, are not fully under an individual's control and may constitute risk factors for health problems.^28^ For example, Black men in disadvantaged neighborhoods without markets and restaurants providing nutritious and affordable foods and beverages, with few parks or other places to walk, and with high noise pollution are less likely to eat healthily, engage in physical activities, and have good quality sleep.^29^ In such instances, maintaining brain health is not completely under their control.

Within the past several decades, social determinants have been posited as a plausible explanation for most health disparities. Yet researchers have only begun to scratch the surface of the crucial components of Black men's brain health, including how social determinants interact with genes, the immune system, and neurological changes.^30^ The intricacy of social determinants lies in the fact that their impact can amass over a lifetime, causing fluctuation in brain health trajectories across one's life course.^31^ Discussion about Black men's brain health and dementia research on Black men must center on the relationship among social determinants that are beyond the control of Black men; individual‐level interventions such as physical activity, sleep, and healthy eating to improve brain health; the extent to which such interventions are under an individual's control; and what supports are needed to help Black men make and sustain modifications to their lifestyles.

Empucate International, a nonprofit organization based in Lexington, Kentucky, works with researchers to demonstrate how community health programs can center social determinants and individual‐level interventions. In collaboration with researchers such as myself, Empucate provides older Black immigrants and resettled refugees, many from the Democratic Republic of the Congo, with plots of land to cultivate food crops, teaches them how to cook and eat healthy foods, and provides opportunities for participation in studies concerning risk for Alzheimer disease and related dementias.^32^ Study findings are shared with community members, local council officials, and landowners who donate their lands to the farmers, focusing on opportunities for policy changes to improve community health. This type of community‐based research holds potential to shape narratives surrounding Black men's brain health, by demonstrating how health interventions can be closely connected to people's interests and to creativity and social connection.

## Emphasis on Strength‐Based Narratives

This example also gives a sense of how it can be beneficial for researchers to shift from deficit‐based approaches that focus on the challenges and barriers Black men face to a strength‐based (or assets‐based) approach that emphasizes their inherent abilities, their skills, and their unique experiences and recognizes their potential for growth and positive change. Strength‐based narratives use language that empowers people and reinforces their sense of agency and control in their own lives—without suggesting that “resilience” alone is a magic remedy.^33^ Researchers can learn to use words and phrases emphasizing strengths and opportunities, such as “community oriented,” “role models,” and “resourceful,” that offer a more nuanced understanding of how Black men can identify and build on their strengths, as a contrast to narratives that depict Black men as victims or without agency.

Critically, a strength‐based narrative recognizes the importance of community support in promoting Black men's brain health. It encourages the development of supportive networks and social connectedness while acknowledging the strengths and successes of Black men as individuals and within their communities. For example, the BMBH project uplifts Black men with positive representations while raising awareness and understanding of Black men's brain health, cognitive aging, and Alzheimer disease and related dementias. Another example is the Louisville, Kentucky, chapter of the African American Male Wellness Agency, led by Kamari Wooten. AAMWA, for which I am a volunteer, leverages community‐wide support to provide Black men with access to health resources, hosting events, such as an annual 5K run, health education, and health screening.

By highlighting the strengths of Black men, researchers can promote a sense of empowerment and agency, which can lead to positive health outcomes and inspire greater engagement with and participation in brain‐health research and greater use of health services. A strength‐based approach can challenge negative stereotypes and stigmas that contribute to health disparities for Black men.^34^ Further, it can promote greater understanding of, empathy about, and social and cultural awareness of dementia.

## Conducting Life‐Course Research with Black Men

The life‐course perspective should guide efforts to understand environmental, social, and genetics factors as they affect Black men's brain health. In gerontology research, the life‐course perspective considers how the experience of individuals is shaped by social and cultural factors over the life course. Although aging happens over time, as yet, only a few researchers employ life‐course approaches to study brain health and dementia in Black men; studies of non‐Hispanic White men provide most of what we know about dementia in men. Understanding the key pathways to brain health—immunological, genetic, and neural—and the most critical periods of exposure to dementia risk factors across the life course will help us design and prioritize efforts to reduce racial disparities in dementia. Using the life‐course perspective to examine brain health acknowledges the complexity of development and health and provides a framework by which to recognize the impact early exposures to risk factors have on brain health in later life.^35^

Diversity in dementia research teams and among study participants is crucial to ensure that dementia research is culturally sensitive and relevant to the experiences of Black men. Research teams focused on dementia in Black men should include Black men, including team members who live in majority‐Black neighborhoods and have similar socioeconomic backgrounds to study participants. This can help ensure that a study design proceeds from commitments to cultural humility and sensitivity and that research questions and other aspects of a study are culturally appropriate. The work I have been involved with as part of the Health and Aging from Minority Perspectives Lab embodies the inclusion of Black men as research collaborators. We seek out Black male leaders and Black‐male‐led community organizations as research collaborators and community‐action board members. For example, Raymond Jetson, the founder of Aging While Black, serves as a consultant on our National Institute on Aging‐funded research project, Early Characterization of Cognitive Status and AD Risk in African American Men. Through these collaborations, we maintain diversity in research and connect with Black men interested in participating in dementia research studies.^36^

Furthermore, research on Black men should incorporate culturally relevant measures and programs. For example, the Black Men's Health Project,^37^ led by a coalition of Black male researchers in the United States, promotes better understanding of lived experiences of Black men while working toward developing programs to improve the health and well‐being of this population. Including culturally relevant measures and programs in dementia research can help ensure that the study outcomes are relevant and meaningful to Black men. To develop culturally appropriate measures and tools in collaboration with Black men, researchers can draw on the example of Alter, a program that demonstrates how community organizations and their leaders can advance dementia research by providing valuable insight into cultural practices and beliefs.^38^ Led by nurse and gerontologist Fayron Epps, Alter collaborates with faith‐based organizations in African American communities to create tailored activities to help congregations become friendly, inclusive places for people with dementia and to study these activities. Working with Black men in research projects aimed at improving their brain health or their well‐being requires participant‐centered approaches to engage with these men and ensure that their voices are heard in the research. Steps include involving participants in study design and implementation, providing appropriate compensation for participation, and making the study accessible and convenient. Additionally, maintaining diversity in dementia research requires that the research team disseminate results back to the Black men in a culturally sensitive and appropriate manner. This can help build trust and promote participation in future research studies.

## Ensuring Access to and Promoting Utilization of Health Care Services

Another important aspect of charting a new frontier for Black men's brain health is understanding that their access to health care is not just a matter of the availability and utilization of health care services but also of their experience of accessing and navigating the health care system. Compared to other groups, Black men are less likely to engage in health‐seeking behaviors and more likely to underutilize health care services, mainly due to mistrust of the medical system, the presence of culturally insensitive care, and biased care providers.^39^ Underutilization of health care services by Black men has also been linked to whether they are insured, to how they prioritize health care among their responsibilities, and to structural and systemic factors such as residential segregation and limited transportation options.^40^ Additionally, underutilization of public programs such as Medicare is evident among eligible Black men,^41^ exacerbating health disparities and limiting their access to preventive care and early detection of dementia.

While there is a tendency to assume that Black men's unhealthy behaviors drive health disparities, efforts to chart a new frontier in Black men's brain health to improve outcomes and reduce disparities must recognize how context shapes the health behavior of this population. Therefore, researchers must show how policies that increase access to and use of health services, such as policies that promote culturally sensitive and affordable health care, can help improve health outcomes in Black men.

## Toward Brain‐Health Equity

Black men, like all individuals, have unique health needs and experiences. Rather than perpetuating harmful stereotypes about Black men's brain health and putting the sole responsibility for brain health on individuals, researchers must strive to address determinants of health, highlight ways to improve access to health care and preventive services, and promote culturally appropriate brain health and dementia education among Black men.

By involving Black men in the dementia research process, researchers can better understand their experiences and needs and ultimately improve the brain‐health outcomes for this population. To chart new frontiers for Black men's brain health and achieve brain‐health equity, researchers must employ a comprehensive approach that identifies and combats systemic barriers to well‐being and promotes awareness among this population of pathways to greater cognitive health.

## Acknowledgments

I was supported by the National Institute on Aging (R00AG078286), an Alzheimer's Association Research Fellowship Grant (23AARFD‐1029261), a Michigan Center for Contextual Factors in Alzheimer's Disease enrichment grant, and the National Institutes of Minority Health and Health Disparities under UK ASCEND (Achieving Success in Community‐Engaged research to elimiNate Disparities) grant (P50MD019476).
